# PPARγ Agonism Modulates Synovial Macrophage and Cartilage Responses in an Equine Model of Synovial Inflammation—Implications for Joint Therapy

**DOI:** 10.3390/biom15091267

**Published:** 2025-09-01

**Authors:** Slàine F. Chaimbeul, Nubia N. P. Rodrigues, Danny D. Thurston, Kirsten E. Scoggin, Jennifer Janes, Cale A. Jacobs, James N. MacLeod, Austin V. Stone, Bruno C. Menarim

**Affiliations:** 1 Department of Veterinary Clinical Sciences, University of Copenhagen, 2630 Taastrup, Denmark; 2 Faculdade de Medicina Veterinária e Zootecnia, Universidade São Paulo, São Paulo 05508-270, SP, Brazil; 3 Rood and Riddle Equine Hospital, Saratoga, NY 12866, USA; 4 Gluck Equine Research Center, Department of Veterinary Sciences, University of Kentucky, Lexington, KY 40506, USA; 5 Veterinary Diagnostic Laboratory, Department of Veterinary Sciences, University of Kentucky, Lexington, KY 40506, USA; 6 Department of Orthopedic Surgery, College of Medicine, Harvard University, Boston, MA 02138, USA; 7 Department of Orthopedic Surgery, College of Medicine, University of Kentucky, Lexington, KY 40506, USA

**Keywords:** joint disease, inflammation resolution, treatment, osteoarthritis

## Abstract

Synovitis resolution is critical for joint homeostasis and prevents the progression of osteoarthritis (OA). Treatments like NSAIDs and intra-articular corticosteroids relieve symptoms by blocking pro-inflammatory mediators, but also impair the production of pro-resolving mediators, contributing to the likelihood of chronic synovitis. PPARγ signaling is an essential mechanism of synovitis resolution, which is decreased in OA tissues. To evaluate the potential of PPARγ agonists to promote pro-resolving pathways, equine macrophages cultured in autologous, normal, or inflamed synovial fluid (*n* = 10 horses) were treated with pioglitazone, geraniol, or both. Treatments modulated patterns of gene expression, increasing the expression of early drivers of resolution *RELB* and *IL6*, followed by increased *NRF2* and *PPARGC1A* expression. Concentrations of TNF-α in conditioned synovial fluid significantly decreased as an early response to treatment, while IL10 concentrations also declined over time, suggesting increased tolerance to inflammatory stimuli and decreased compensatory feedback. Using an equine model of synovitis, intra-articular delivery of pioglitazone (*n* = 3 horses) or geraniol (*n* = 4 horses) was associated with decreased markers of synovium inflammation (geraniol) and enhanced cartilage proteoglycan preservation (geraniol and pioglitazone). In this small cohort of horses, no systemic or articular side effects were observed. Further studies optimizing treatment doses and regimens for intra-articular PPARγ agonism as a pro-resolving OA therapy are warranted.

## 1. Introduction

Osteoarthritis (OA) is a debilitating condition that affects over 30% of the global population and is the second-most costly health condition to the American Health Care system [1,2,3]. OA is also the leading cause of lameness and early retirement in equine athletes, translating into the leading cause of financial losses in the equine industry [4,5]. Chronic synovitis is a common feature of all arthritic conditions and eventually leads to OA [6,7,8,9]. Despite therapeutic advances, there remains a need to identify more effective ways to treat synovitis, restore joint homeostasis, and halt OA progression. Current therapies are largely based on anti-inflammatory principles, thus blocking pro-inflammatory mediators such as PGE_2_ and NF-κB signaling [10,11,12,13]. However, these same mediators are also required for the natural endogenous resolution of inflammation [14,15]. Therefore, the use of anti-inflammatory drugs to inhibit pro-inflammatory mediators can impair cellular mechanisms of timely resolution and thereby increase the susceptibility to chronic low-grade synovitis seen in OA [16,17]. One way to avoid these and other side effects from these medications, instead of blocking inflammation, is to stimulate endogenous resolution. Orthobiological treatments offer pro-resolving benefits compared to anti-inflammatory drugs; however, related improvements are inconsistent and often short-lasting [18,19,20,21,22,23]. New therapies capable of resolving joint inflammation while preserving homeostatic mechanisms are a critical need.

Successful treatment of OA is limited by a poor understanding of the central drivers of synovial inflammation resolution—macrophages. While synovial macrophages can incite inflammation, they are the cornerstone of resolution through a process that requires a fine balance of pro- and anti-inflammatory mechanisms counteracting damage, driving tissue repair and recovery of joint health [24,25,26,27,28]. OA results, at least partially, from the impaired ability of synovial macrophages to resolve inflammation [28]. We recently showed that injecting autologous macrophages from bone marrow mononuclear cells (BMNCs) into inflamed joints results in marked inflammation resolution [27]. The clinical outcomes from horses and people with OA treated with BMNCs are comparable to those of corticosteroids, yet the benefits are more consistent and long-lasting, with the advantage of no side effects [29,30,31]. To uncover mechanisms by which BMNCs induce resolution, we profiled the molecular response of BMNCs to joint inflammation. BMNCs mediate resolution through a short pro-inflammatory response that triggers macrophage proliferation and synthesis of biolipids from the mevalonate pathway that enhance agonism of peroxisome proliferator-activated receptor gamma (PPARγ) [32]. PPARγ signaling is crucial to downregulate canonical NF-κB signaling, adjust mitochondria metabolism to decrease inflammatory oxidative stress, and increase efferocytosis and the production of pro-resolving cytokines and growth factors (e.g., IL10 and IGF1) [33]. Logically, PPARγ signaling is vital for the metabolism of synovial macrophages and chondrocytes, but is decreased in osteoarthritic tissues, further disrupting joint homeostasis [34,35,36,37,38]. As a matter of fact, PPARγ knockout induces spontaneous premature OA, and PPARγ agonism has marked cartilage-preserving effects [34,35,36,37,38].

Therapeutic enhancement of PPARγ signaling provides a unique opportunity to induce resolution of synovial inflammation. Lipid mediators derived from the mevalonate pathway are essential for effective homeostasis of the innate immunity, and some of them are PPARγ agonists [39,40,41,42]. Overall, their lack or deficiency can cause severe inflammatory conditions, but their supplementation reestablishes homeostasis [43,44]. Synthesis of such lipids by synovial macrophages is decreased during joint inflammation [32]. The use of geraniol, a mevalonate-derived isoprenoid, to treat arthropathies has just recently been explored, with initial studies in mice with promising results [45]. Treatment with thiazolidinediones, such as pioglitazone, to enhance PPARγ signaling provides remarkable pro-resolving and cartilage-saving effects in dogs and rodents. However, the systemic side effects of oral thiazolidinediones prevented further research in this field [35,36]. Intra-articular delivery of either thiazolidinediones or mevalonate isoprenoids by joint injection can reduce the potential systemic side effects. The aim of this pilot study was to assess the pro-resolving effects of pioglitazone and geraniol in equine models of synovitis, both in vitro and in vivo. We hypothesized that both molecules would induce a pro-resolving response comparable to BMNCs.

## 2. Materials and Methods

### 2.1. Study Design

#### 2.1.1. In Vitro Study

**Figure 1:**
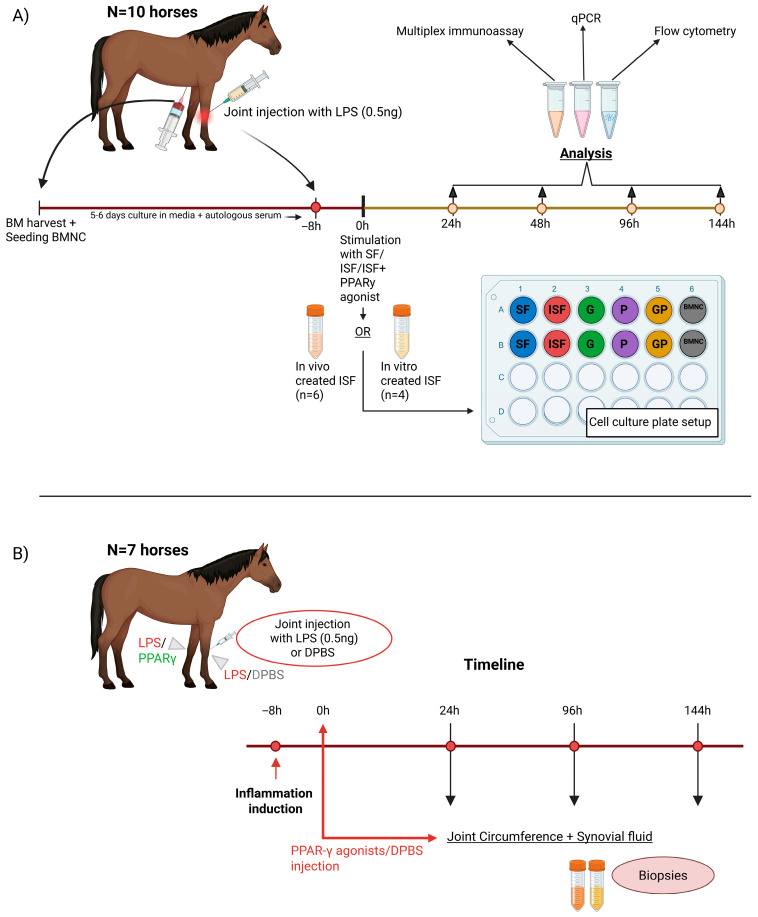
(**A**) In vitro study design. Bone marrow was harvested from 10 horses and cultured for 5–6 days in culture media. Synovitis was induced with lipopolysaccharide (LPS) (0.5 ng/joint, *n* = 6 horses), and inflamed synovial fluid (ISF) was collected after 8 h at the peak of the inflammatory reaction. Alternatively, normal synovial fluid (SF) was spiked with TNF-α, IL1β, and LPS (see Methods) to create an ISF equivalent. A schematic denoting the culture plate setup with different treatments (G, P, and GP) and controls (SF, ISF and BMNCs). Cells and conditioned media (ISF/SF) were harvested and assayed at 24 h, 48 h, 96 h, and 144 h. (**B**) In vivo study design. Study design showing the timing of LPS injections, treatment with PPARγ agonists (geraniol or pioglitazone). and sample collection. SF = normal synovial fluid, ISF = inflamed synovial fluid, G = geraniol (17 ng/mL) in ISF, P = pioglitazone (20 ng/mL) in ISF, GP = combination of geraniol and pioglitazone in ISF, BMNCs = bone-marrow mononuclear cells in ISF.

Ten skeletally mature thoroughbred horses (3–7 years old, median 4 years, 8 castrated males, 2 females) free of systemic inflammation or OA in the studied joints were used under IACUC (***2022-3992***) approval and oversight. General and musculoskeletal health was confirmed by clinical, hematological, and orthopedic evaluations, including diagnostic imaging. Sternal bone marrow aspirates were obtained for bone marrow mononuclear cell (BMNC) isolation and culture of bone marrow-derived macrophages (BMDMs). BMDMs were assayed in autologous normal (SF) or inflamed synovial fluid (ISF) following native cell depletion (Figure 1). To produce ISF with more homogeneous inflammation than could be acquired from naturally occurring OA at varying stages of disease, ISF was produced by a model of lipopolysaccharide (LPS)-induced synovitis in both radiocarpal and middle-carpal joints [46] (*n* = 6 horses) or treatment of SF with LPS and cytokines, as detailed below (*n* = 4 horses—see details and rationale below). SF was collected from healthy joints (metacarpophalangeal, metatarsophalangeal, and/or femorotibial joints). After reaching 70% confluence in culture media, BMDMs from each horse were assayed independently in neat (100%) autologous SF or ISF with or without the PPARγ agonists geraniol (G), pioglitazone (P), or a combination of both (GP) and harvested at 24, 48, 96, and 144 h. Controls included SF, ISF with no treatment, and ISF with a dose of BMNCs known to induce inflammation resolution [27,47]. At each time point, macrophages were assayed for gene expression and flow cytometry to characterize treatment responses. Real-time quantitative PCR (qPCR) was used to assess the expression of genes previously associated with inflammation and inflammation resolution and PPARγ signaling, including mitochondrial function and biogenesis. Flow cytometry was used to assess mitochondrial biogenesis and production of reactive oxygen species (ROS). At the same time points, cytokine and growth factor secretion were quantified in conditioned synovial fluid.

#### 2.1.2. In Vivo Study

To assess the effects of these PPARγ agonists in vivo, seven skeletally mature Thoroughbred horses (3–9 years old, median 6 years, 3 castrated males, 4 females) free of systemic inflammation or OA in the studied joints were subjected to the same model of LPS-induced synovitis in both radiocarpal joints. After 8 h, at peak inflammation, for each horse one radiocarpal joint was treated with geraniol (*n* = 4 horses, 2 males, and 2 females) or pioglitazone (*n* = 3 horses, 1 male, and 2 females), while the contralateral joint was treated with DPBS as control. Synovial fluid was collected at 24, 96, and 144 h following treatment. After obtaining the last synovial fluid sample, horses were humanely euthanized. Macroscopic assessment of the joints was performed, and synovial membrane and cartilage samples were subjected to histology.

### 2.2. BMNC Isolation and Macrophage Culture

Two bone marrow aspirates (25 mL/each) were collected from the 4th and 5th sternebrae of each horse. Using an aseptic technique, bone marrow was aspirated into 60 mL syringes prefilled with 15,000 IU heparin (083259, Covetrus, Lexingotn, KY, USA) in 25 mL sterile DPBS [27,48]. BMNCs were isolated by density gradient centrifugation with Ficoll-Paque (GE Healthcare, Waukesha, WI, USA) medium and washed with DPBS twice before removing the remaining red blood cells with a red cell lysis buffer for 20 min [27]. After centrifugation, the BMNC pellet was washed twice with DPBS and assessed for concentration and viability using trypan blue staining. Peripheral blood (300 mL/horse) was collected from jugular venipuncture to isolate autologous serum for cell cultures.

For the generation of BMDMs, BMNCs were seeded in 24-well culture plates (1.5 × 10^6^ viable cells/50 µL DPBS/well) to generate duplicate samples for each of the six experimental groups (Figure 1). Each well was covered with IMDM (Corning, Tewksbury, MA, USA) containing 25% autologous serum, 25 ng/mL M-CSF, 1% penicillin–streptomycin, and 0.1% gentamicin. The remaining BMNCs were cryopreserved in 90% fetal bovine serum (FBS) containing 10% dimethyl sulfoxide (DMSO, Sigma, Norwood, OH, USA) at 25–50 × 10^6^ cells/mL until further use. Culture media were not exchanged, but 200 µL was added to each well every 48 h.

### 2.3. Induction of Synovitis and Processing of Inflamed Synovial Fluid

To produce ISF for in vitro experiments, an in vivo model of synovitis was created in 6/10 horses by injection of 0.5 ng LPS (L2637, Sigma, Norwood, OH, USA) suspended in 2 mL DPBS into each middle carpal and radiocarpal joint (Figure 1). Eight hours following the induction of synovitis, at peak inflammation, ISF was aspirated from the referred joints using an aseptic technique and pooled. SF was harvested from metacarpophalangeal, metatarsophalangeal, and/or femorotibial joints and pooled. After harvest of synovial fluid, all horses were humanely euthanized under American Veterinary Medical Association (AVMA) guidelines due to reasons unrelated to the current study.

Preliminary results from the first 6 horses suggested there were biological differences between treatments, although without statistical support. Therefore, to increase the magnitude of the response of BMDMs to the inflammatory stimulus and our likelihood of identifying a response to treatment, for the remaining four horses, ISF was created in vitro by the addition of IL1β (1 ng/mL, R&D Systems, Minneapolis, MN, USA), LPS (0.1 ng/mL), and TNF-α (0.028 ng/mL, R&D Systems, Minneapolis, MN, USA) to SF from radiocarpal and middle carpal joints. Doses for these mediators were chosen to duplicate the concentrations identified in ISF from the in vivo model [27,47]. For both models of ISF, following harvest, synovial fluid was centrifuged at 5000× *g* for 30 min at 4 °C to deplete cells. The cell-free supernatant was then recovered, and antibiotics (1% penicillin–streptomycin, 0.1% gentamicin) were added before using it as cell culture media.

### 2.4. Inflammatory Challenge and Treatment with PPARγ Agonists

Following growth in culture media for 5–7 days to reach ~80% confluence of BMDMs, each well was gently washed with warm DPBS (37 °C) to remove the unattached progenitors from BMNCs. Macrophages were challenged (independent wells, not pooled) in neat (100%) autologous SF, ISF, or ISF + treatment. BMDMs were stimulated with 400 µL SF, ISF, ISF + geraniol (G, Sigma, St. Louis, MO, USA), ISF + pioglitazone (P Sigma, St. Louis, MO, USA), ISF + geraniol + pioglitazone (GP), or ISF + 2 × 10^6^ autologous BMNCs/well. Doses of the PPARγ agonists geraniol (17 ng/mL) and pioglitazone (20 ng/mL) were identified in previous studies [45,49], and a titration assay using the cells of four different horses verified patterns of gene expression and cell morphology treated with a range of doses (geraniol—13, 17, and 21 ng/mL; pioglitazone—20, 30, and 40 ng/mL) to establish dose response. In the combination treatment, GP, both geraniol and pioglitazone were added in the same doses as used individually. In the BMNC-treated group, 2 × 10^6^ BMNCs were added to each well along with 400 uL ISF. Every 48 h, cultures were supplemented with 200 µL of their corresponding media, but no further cells were added to the BMNC wells. All treatments were performed in duplicate wells and harvested at 24, 48, 96, and 144 h for flow cytometry, mRNA extraction, and cytokine/chemokine quantification.

### 2.5. Cell Harvesting and Processing

Following removal of conditioned SF or ISF for immunoassays, cell culture wells were washed in 1 mL of warm DPBS (37 °C) to remove residual synovial fluid and cells were detached in 2 mL cold 10 mM EDTA in DPBS for 10 min. Cells were gently pipetted to release adherent cells, and the cell suspension transferred to Protein LoBind microfuge tubes, centrifuged (1500× *g*; 4 min; 4 °C), and washed in DPBS. Cell pellets from one well of each treatment group were used for flow cytometry, while those from the duplicate well were lysed in guanidinium chloride–phenol (Trizol^®^, Invitrogen, Carlsbad, CA, USA).

### 2.6. Gene Expression

RNA was purified with on-column DNase digest (DirectZol™ RNA microprep kit, R2061, Zymo Research, Irvine, CA, USA), quantified, and stored at −20 °C. Expression of genes associated with inflammation (*IL1B*, *IL6*), inflammation resolution (*IL10*, *RELB*), and PPARγ signaling (*PPARG*, *PPARGC1A*), including mitochondrial function and biogenesis (*NRF1, NRF2, TFAM*), were assessed relative to the expression of internal controls (*GAPDH, ACTB*) using RT-QPCR. Briefly, mRNA was reverse-transcribed and RT-QPCR was performed using 5 µL diluted cDNA (2 ng/µL), 0.25 µL of each primer (forward and reverse, 100 ng/µL), 6.25 µL PowerUp SYBR Green mix (#A25741, Thermo Fisher Scientific, Vilniaus, Lithuania), and 0.75 µL ddH_2_O. All primers (Supplementary Table S1) were custom-designed, commercially synthesized (Invitrogen, Carlsbad, CA, USA), and subjected to efficiency assessment. Reactions were performed in duplicate on a ViiA7 Real-Time PCR system (Applied Biosystems, Thermo Fisher Scientific, Lithuania). Expression (mRNA transcripts) of the selected genes was determined relative to the geometric means of β-actin (ACTB) and GAPDH as the reference transcripts (internal controls). All reactions were automatically pipetted using epMotion 5070 automated pipetting systems (Eppendorf, Hamburg, Germany). Fold changes were determined by the ΔΔCT method [50]. Due to limited RNA availability, data for the expression of some genes were limited to seven horses for some time points. For the total of 36 datasets (9 genes × 4 time points), 8 included 10 horses, 17 included 9 horses, 7 included 8 horses, and only 4 were restricted to 7 horses.

### 2.7. Flow Cytometry

Macrophages from four horses (castrated males) at all time points were subjected to flow cytometry analysis. Cells were stained in 300 µL DPBS containing nonyl acridine orange for assessment of mitochondrial mass (2.5 µM, A1372, Invitrogen, Eugene, OR, USA) [51], CellRox deep red for quantifying reactive oxygen species (ROS) production (manufacturer recommendations, C10422, Invitrogen, Eugene, OR, USA), Zombie Violet for viability (as per manufacturer recommendation, 423114, BioLegend, San Diego, CA, USA) for 30 min at 37 °C, 5% CO_2_. After staining, cells were washed twice in DPBS, centrifuged at 1500 g for 3 min at 22 °C, resuspended in 300 µL DPBS, and kept cold until analyzed (BD Biosciences FAC Symphony, Columbus, NE, USA). Flow cytometry data were processed—1st—cell size, 2nd—singlets, 3rd—cell viability—and evaluated using median fluorescence intensity (MdFI), with FlowJo software v.10.10 (BD Life Sciences). All flow cytometry data were acquired on cells stimulated with ISF produced in vivo (*n* = 4).

### 2.8. Joint Treatment and Synovial Fluid Sampling

Using an aseptic technique, one inflamed radiocarpal joint was treated with 170 ng geraniol–2 mL DPBS (*n* = 4 horses) or 200 ng pioglitazone–2 mL DPBS (*n* = 3 horses), while the contralateral joint was treated with 2 mL DPBS as a control. The intra-articular dose of each molecule was selected to approximate the same concentration tested in vitro, taking into consideration the expected volume of synovial fluid for a radiocarpal joint (10 mL). At 24, 96, and 144 h following treatment, synovial fluid samples were collected and centrifuged (1500× *g* × 10 min at 4 °C) and the cell-free supernatant frozen at −80 °C for batch processing and cytokine/chemokine quantification, as described below.

### 2.9. Multiplex Immunoassay

Synovial fluid samples were processed using low-retention pipette tips and stored in Protein LoBind microfuge tubes. Conditioned synovial fluid from each treatment group was collected, centrifuged (1500× *g* × 10 min at 4 °C), and the cell-free supernatant frozen at −80 °C for batch processing and cytokine/chemokine quantification. Further, synovial fluid samples were hyaluronidase-digested (100 IU/mL testicular hyaluronidase in 0.05 M acetate buffer pH 4.5, LS005474; Worthington Biochemical, Lakewood, NJ, USA) using 10 µL of hyaluronidase solution per 200 µL of synovial fluid and incubated for 30 min at 37 °C. Samples were then centrifuged for 10 min at 12,000× *g* and 4 °C to remove any particulate matter, and the supernatant was recovered [27,52]. Using the Miliplex equine inflammation panel (EQCTTMAG-93K; Milliplex MAP Equine chemokine/cytokine, MilliporeSigma, Burlington, MA, USA), concentrations of IL1α, IL1β, IL6, IL10, TNF-α, SDF1, and MCP1 were quantified. The minimum detection (MinDC + 2SD) values for the cytokines were as follows: IL1α (39.1 pg/mL), IL1β (29.3 pg/mL), IL6 (1.4 pg/mL), IL10 (62.5 pg/mL), TNF-α (5.2 pg/mL), SDF1 (55.8 pg/mL), and MCP1 (399 pg/mL). The standard curve ranges for the cytokines were: IL1α (59–60,000 pg/mL), IL1β (29–30,000 pg/mL), IL6 (15–15,000 pg/mL), IL10 (49–50,000 pg/mL), TNF-α (4–4000 pg/mL), SDF1 (97.7–100,000 pg/mL), and MCP1 (342–350,000 pg/mL).

### 2.10. Histology

Following euthanasia, joints were evaluated for macroscopic signs of inflammation (e.g., synovial hyperemia and hematomas), as well as intra-articular and periarticular hemorrhage and edema. Two synovial membrane biopsies from each joint were obtained using a 6 mm dermal biopsy punch at sites adjacent to where radiocarpal joints most often show cartilage degeneration [53]. Biopsies were fixed in AZF Fixative (1009B; Newcomer Supply, Middleton, WI, USA) for 24 h, rinsed, and stored in PBS at 4 °C until processing for histology. Paraffin-embedded samples were sectioned at 5 µm and hematoxylin and eosin–stained. Gross pathology and synovial membrane and cartilage histology were blindly scored by a board-certified veterinary anatomic pathologist. Synovial membrane histology was assessed on H&E-stained sections using the Osteoarthritis Research Society International (OARSI) histopathology scoring system while ignoring synovial fibrosis due to the acute nature of the model. Cartilage samples from the experimental joints were available for 1/4 of the horses treated with geraniol and all three horses treated with pioglitazone. Cartilage samples were fixed as described and comparatively assessed between treated and control joints for the extension and intensity of Safranin O staining.

### 2.11. Statistical Analysis

Data analysis was performed with assistance from the Applied Statistics Lab at the University of Kentucky. Data from the in vitro study were Box–Cox-transformed to allow the use of parametric statistical measures and cluster analysis of datasets generated with the two different sources of ISF (in vivo model vs. in vitro spike). Data generated with the different ISF models were analyzed following normalization of each dataset, independently as well as combined, and no differences in the outcome were observed. To assess whether the origin of the ISF model (in vivo vs. in vitro) influenced the response to treatment, a linear mixed-effect model was fitted, and no effect was observed. Therefore, statistical analysis was ultimately performed combining both datasets. Gene expression data for all treatments were normalized to ISF at each time point, and fold-change values were log_10_-transformed for normality. For cytokine values that were below the lower limit of detection (LLOD), 0.5 *LLOD imputed values were used for statistical analysis. Concentrations were subsequently log_10_-transformed to normalize data distribution prior to statistical testing. Flow cytometry data were also log_10_-transformed before analysis. Post hoc Dunnett’s tests were used to adjust for multiple comparisons. Data were analyzed in GraphPad Prism (version 10.2.0) using either a mixed-effect model or one-way ANOVA, depending on the presence of missing values. Significance was set at *p* < 0.05. Histological data were analyzed descriptively, since the OARSI scoring system failed to capture the main differences observed between treated and untreated joints and the number of cartilage samples was very low.

## 3. Results

### 3.1. Cell Culture Behavior

**Figure 2:**
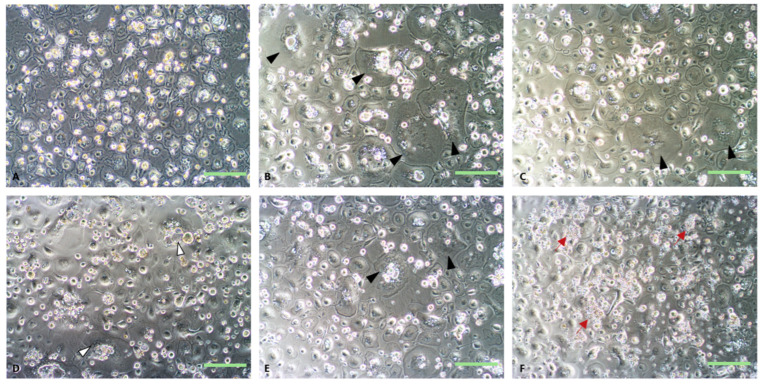
Equine BMDMs following 96 h of culture in autologous normal (SF (**A**)) and inflamed synovial fluid (ISF (**B**)) +/− the addition of the PPARγ agonists geraniol (**C**), pioglitazone (**D**), geraniol + pioglitazone (**E**), and BMNCs (**F**). Note differences in cell size, morphology ((**A**) vs. (**B**–**E**) and (**B**) vs. (**C**–**F**)), multinucleation (black arrowheads), intracellular granularity ((**D**)—white arrowheads) and intercellular gaps between BMDMs in SF and ISF. Following treatment with ISF + PPARγ agonists, cell morphology features were partially and inconsistently rescued. BMDMs treated with BMNCs exhibited abundant clusters of small mononuclear cells (BMNCs, red arrows), which provided a pool for macrophage self-renewal, with abundant small plastic-adherent macrophages over time. Bars = 200 μm.

BMNC cultures differentiated into confluent BMDM populations. Upon exposure to treatments in synovial fluid (SF, ISF, G, P, GP, and BMNCs), notable variations in cell morphology and size were observed (Figure 2). Cultures in ISF (with/without PPARγ agonists) exhibited increased heterogeneity, higher proliferation rates evidenced by abundant small and non-adherent cell clusters, and higher frequency of large multinucleated cells. These cultures also exhibited more pronounced intercellular gaps and activation features (irregular cell surfaces and intracellular granularity) compared to those cultured in SF (Figure 2B–E, black arrows). Subjectively, the use of PPARγ agonists partially rescued cell morphology features, most noticeably at 96 h, although these findings were inconsistent.

### 3.2. Gene Expression

**Figure 3:**
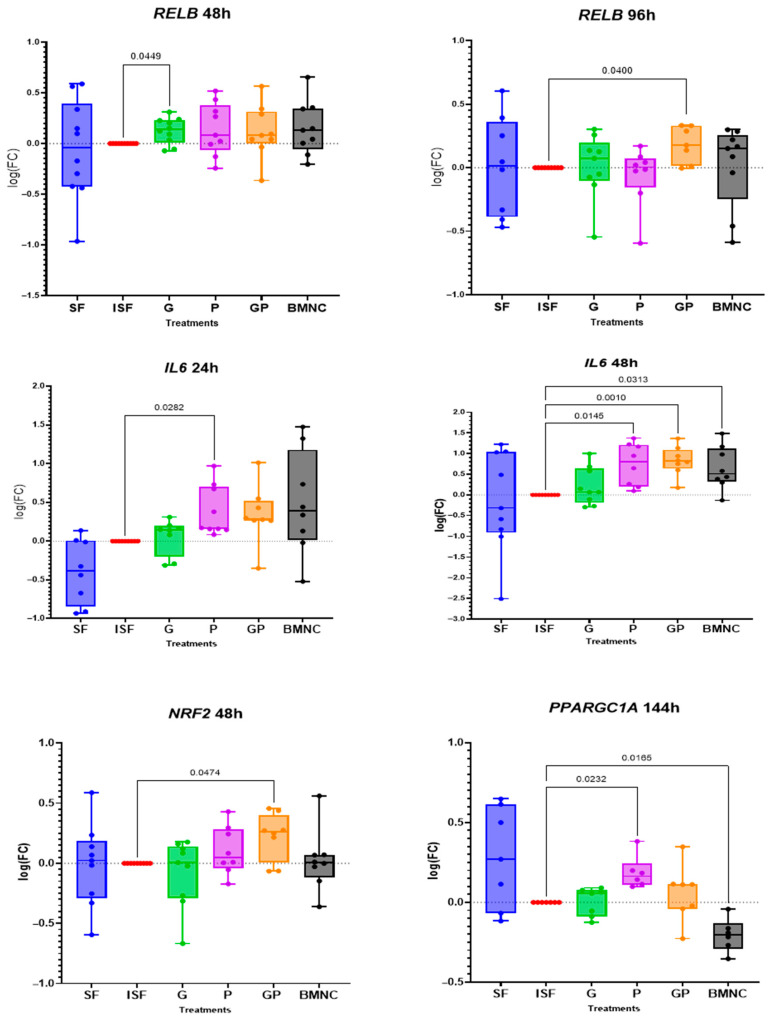
Expression profiles of genes for which a significant effect of treatment was observed. All treatments increased mean *RELB* gene expression comparably to BMNCs at 48 h, but only G (48 h) and GP (96 h) were significant. Early rises in *IL6* gene expression more closely resembled BMNCs in P- (24 h) and GP-treated cells (48 h). Changes in *RELB* and *IL6* expression followed the timeline expected for a pro-resolving response, as previously reported [32]. GP also induced changes in expression of the mitochondria subunit gene *NRF2* (48 h), while P was associated with a higher expression of *PPARGC1A*, the PPARγ coactivation factor. SF = normal synovial fluid (blue), ISF = inflamed synovial fluid (red), G = geraniol (17 ng/mL) in ISF (green), P = pioglitazone (20 ng/mL) in ISF (purple), GP = combination of geraniol and pioglitazone in ISF (orange), BMNCs = bone marrow mononuclear cells in ISF (gray). Data are shown as log10 (fold change (FC)) relative to ISF at the given time point. Box plots display the medians and interquartile ranges, and whiskers denote data ranges.

Treatment-related changes in the expression of genes associated with inflammation resolution were observed for *RELB*, *NRF2*, and *PPARGC1A*, and for *IL6*, which is related to both inflammation and its resolution (Figure 3). Expression of *RELB,* the cornerstone of non-canonical NF-kB signaling during inflammation resolution, was significantly upregulated in response to geraniol at 48 h and by combination of geraniol and pioglitazone at 96 h (Figure S3). *IL6* was upregulated at 24 h in response to pioglitazone and at 48 h in response to both pioglitazone and the combination of geraniol and pioglitazone comparably to BMNCs. Expression of the gene coding for nuclear respiratory factor 2 (*NRF2*), required for mitochondrial biogenesis during inflammation resolution, was upregulated at 48 h in the geraniol + pioglitazone treatment. Finally, expression of *PPARGC1A*, the gene coding for PPARγ coactivator factor 1a and essential for mitochondrial biogenesis, was significantly upregulated in response to pioglitazone at 144 h, while it was downregulated in the BMNC treatment group at the same time point. Additional findings included *IL1B* upregulation in the BMNC-treated group, which was significant at 48 h and 144 h, while IL10 was upregulated in the BMNC-treated group at 144 h (Figures S4 and S5).

### 3.3. Flow Cytometry

**Figure 4:**
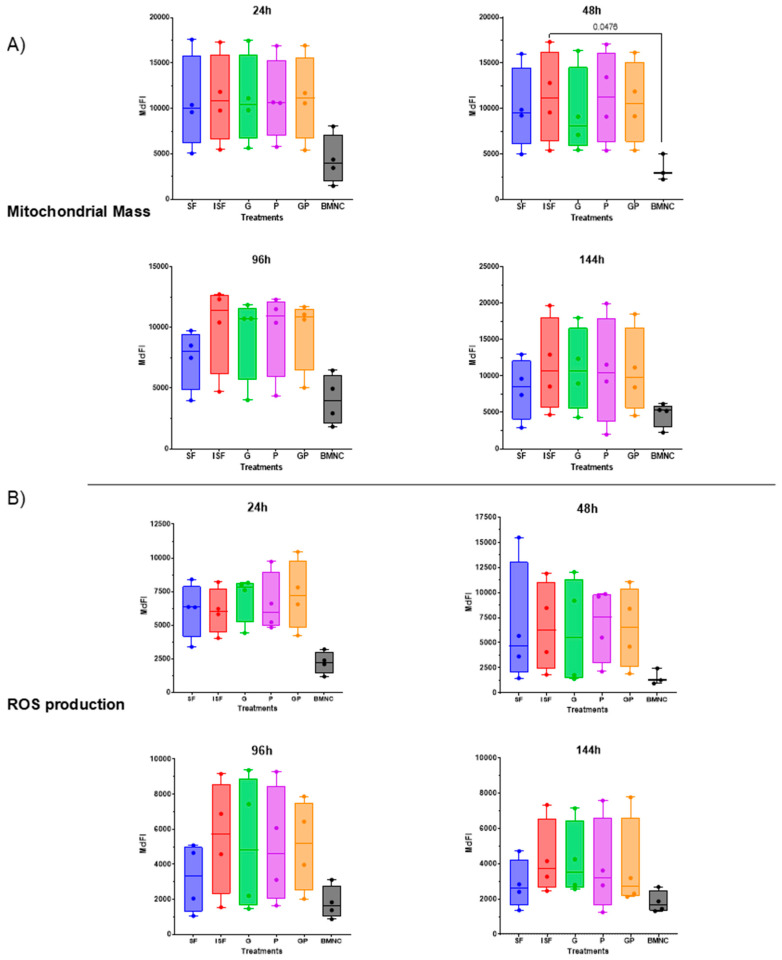
Flow cytometric assessment of (**A**) mitochondrial mass and (**B**) reactive oxygen species (ROS) production. No significant differences were observed between PPARγ-treated cells and ISF at any time point. BMNCs showed lower mitochondrial mass and ROS production, likely due to smaller cells compared with BMDMs. Data presented as raw values for clarity. Box plots display the medians and interquartile ranges, and whiskers denote data ranges. Mitochondrial mass was significantly different from ISF at 48 h (*p* = 0.048). The lower mitochondrial mass of BMNCs is likely due to smaller cells and stage of development compared with BMDMs. SF = normal synovial fluid (blue), ISF = inflamed synovial fluid (red), G = geraniol (17 ng/mL) in ISF (green), P = pioglitazone (20 ng/mL) in ISF (purple), GP = combination of geraniol and pioglitazone in ISF (orange), BMNCs = bone marrow mononuclear cells in ISF (gray). Median fluorescence intensity (MdFI).

The MdFI of nonyl acridine orange, reflecting mitochondrial mass, and CellRox deep red, indicating ROS production, showed no significant differences among the experimental groups at any time point, except for differences in mitochondrial mass between BMNCs and ISF at 48 h (Figure 4). A trend of increased mitochondrial mass and ROS production in response to inflammation was observed at 96 h, although differences between groups failed to reach significance.

### 3.4. Cytokine/Chemokine Quantification

**Figure 5:**
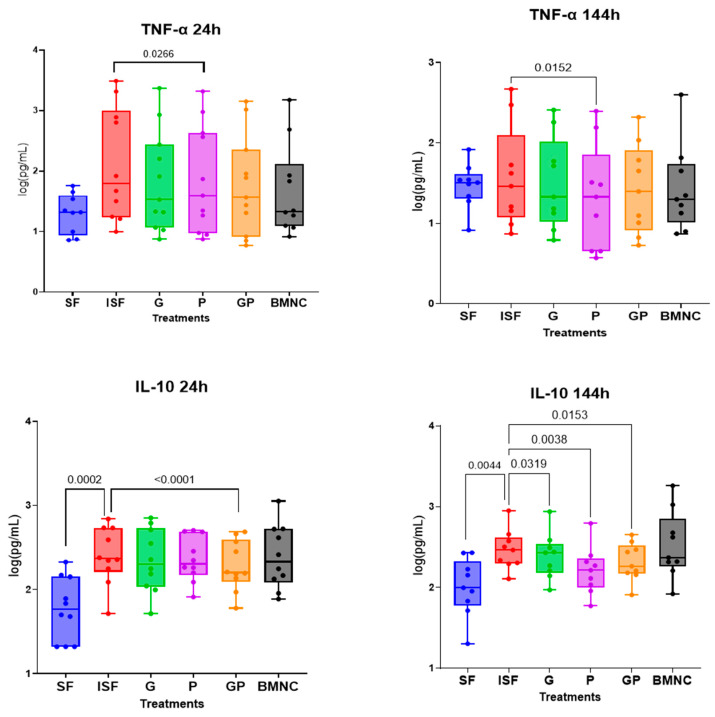
Concentrations of TNF-α and IL10 in conditioned synovial fluid. Significantly decreased synovial fluid concentrations of TNF-α were observed at 24 h for cells treated with inflamed synovial fluid containing pioglitazone or the combination of pioglitazone and geraniol and were comparable to BMNCs. At 144 h, TNF-α concentrations were also lower for cells treated with pioglitazone. On the other hand, an early effect on IL10 concentrations was observed only for geraniol + pioglitazone-treated cells. Nonetheless, at 144 h, conditioned medial from all PPARγ-treated cells exhibited significantly lower concentrations of IL10 than in ISF. SF = normal synovial fluid (blue), ISF = inflamed synovial fluid (red), G = geraniol (17 ng/mL) in ISF (green), P = pioglitazone (20 ng/mL) in ISF (purple), GP = combination of geraniol and pioglitazone in ISF (orange), BMNCs = bone marrow mononuclear cells in ISF (gray). Data are shown as log_10_ (concentration). Box plots display the medians and interquartile ranges, and whiskers denote data ranges.

Cytokine/chemokine concentrations were detectable for five of seven analytes assayed. MCP1 and IL1α were below detection limits for all samples. Treatment with PPARγ agonists was associated with reduced concentrations of TNF-α and IL10 in the conditioned synovial fluid (Figure 5). Significantly decreased synovial fluid concentrations of TNF-α were observed at 24 h for pioglitazone when compared to ISF, comparable to BMNCs. These observations were ultimately (144 h) associated with lower concentrations of IL10 for all treatments. SDF1 concentrations were not different in any PPARγ treatment compared with ISF, but elevated in SF compared with ISF at 96 h and 144 h. Concentrations of IL6 and IL1β were as expected, significantly lower in SF compared to ISF at all time points (*p* < 0.05), but none of the PPARγ treatments or BMNCs were different compared to ISF (Figures S6 and S7). Cytokine data from the in vivo study will be part of future work.

### 3.5. Gross Pathology and Histology

**Figure 6:**
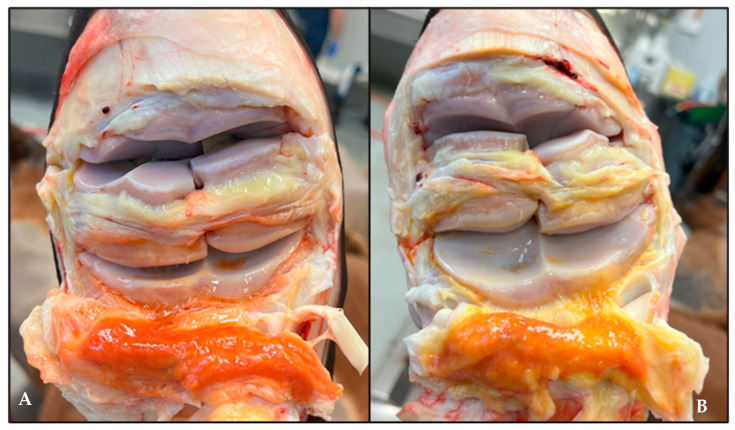
Inflamed joints treated with geraniol (**A**) showed gross improvements in the synovium, characterized by less swelling, congestion, and hemorrhage, compared with joints treated with DPBS (**B**).

**Figure 7:**
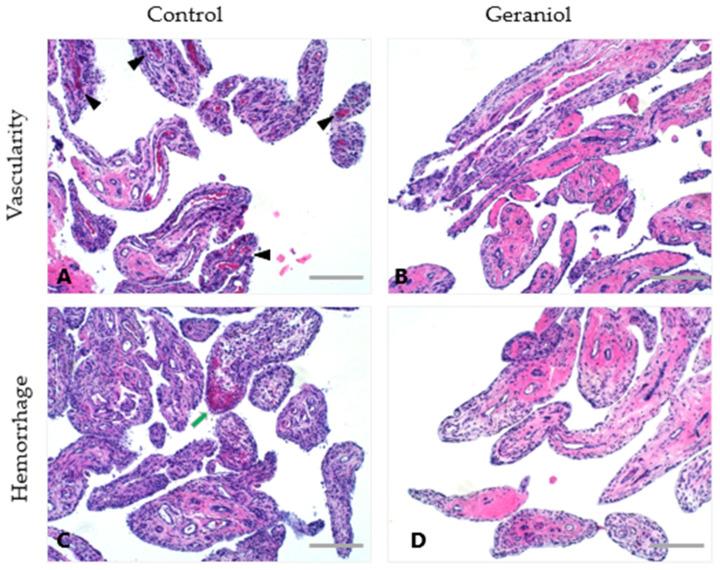
Representative H&E images depicting differences in histologic parameters between inflamed joints treated with geraniol or DPBS. Geraniol-treated joints exhibited lower vascularity (black arrowheads on control samples, (**A**)) and no intimal hemorrhage (green arrow, (**C**)) compared to DPBS-treated joints in 3/4 horses. Bars = 100 μm.

**Figure 8:**
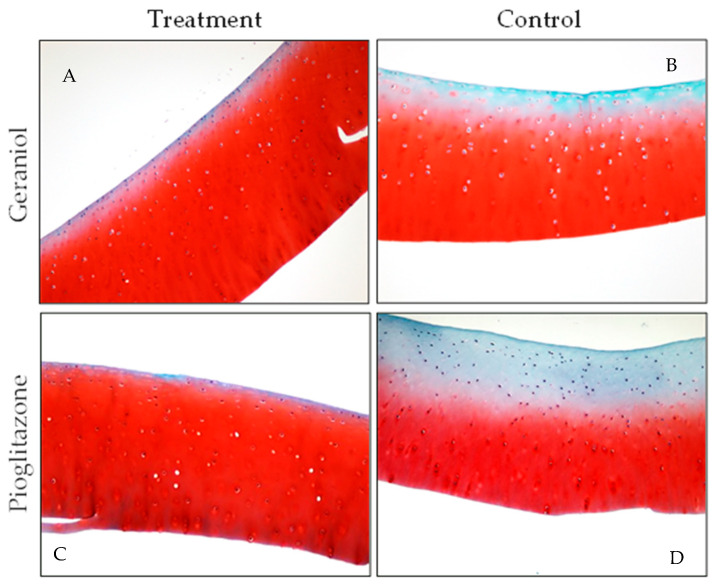
(**A**–**D**) Cartilage from matching joints of two horses. Cartilage from joints treated with either geraniol (*n* = 1) or pioglitazone (*n* = 3) exhibited preserved proteoglycan content, depicted by red staining, while those treated with DPBS showed significant proteoglycan loss from the superficial layer of the cartilage.

For three of four horses in the geraniol-treated group, the pathologist was able to accurately differentiate between geraniol-treated joints and DPBS-treated controls, the latter exhibiting more marked signs of inflammation (Figure 6). No gross or histological effects were observed in the synovia of the three horses treated with pioglitazone. Key histological differences observed between geraniol- and DPBS-treated synovia were reflected by lower vascularity and no intimal hemorrhage, which was evident in DPBS-treated joints in three of four horses (Figure 7). All four cartilage samples from joints treated with either geraniol or pioglitazone exhibited preserved proteoglycan content (Figure 8C,D), while those treated with DPBS showed significant proteoglycan loss from the superficial layer of the cartilage (Figure 8A,B).

## 4. Discussion

This study investigated the pro-resolving effects of two PPARγ agonists, geraniol and pioglitazone, using in vitro and in vivo equine models of synovial inflammation. In our study, the response to pioglitazone and geraniol varied, but yielded measurable homeostatic responses associated with inflammation resolution in both models. In vitro, these responses were characterized by temporal changes in macrophage gene expression for *RELB*, *IL6*, *NRF2*, and *PPARGC1A*, critical regulators of inflammation resolution and related mitochondria biogenesis, at time points crucial for establishing a pro-resolving response [32]. Treatment with these PPARγ agonists was also associated with lower concentrations of TNF-α in conditioned ISF at 24 h for pioglitazone, comparably to BMNCs, which were ultimately associated with decreased concentrations of IL10 for all treatments. Combined differences in cytokine concentrations are suggestive of increased tolerance to inflammatory stimulus and decreased compensatory feedback [54]. In vivo, such findings were associated with improved markers of synovium and/or cartilage inflammation, consistent with previous studies showing protective effects of articular cells and tissues in those treated with pioglitazone or geraniol, although not comparable to BMNC [27,35,45]. No systemic or articular side effects to intra-articular treatment with geraniol or pioglitazone were observed in this small cohort of horses.

While increases in gene and protein expression for IL10 are classically expected during inflammation resolution [27,28], such increases are directly proportional to the expression of so-called pro-inflammatory cytokines such as TNF-α [54]. In our study, the lower production of TNF-α in ISF conditioned by cells treated with the PPARγ agonists suggests increased tolerance to inflammatory insult. This was further supported by the proteoglycan preservation in the cartilage of treated joints. depicted by expression and Changes in the expression of *RELB* and *PPARGC1A* are aligned with our previous findings of macrophage mediated resolution, where *RELB*, *PPARGC1A*, and *PPARG* were among the most upregulated genes in activated pathways during a PPARγ-mediated pro-resolving response [32]. RELB is a cornerstone of non-canonical NF-κB signaling and essential for the development and maturation of cells from the myelomonocytic lineage, which are central players in tissue repair, increased resistance to damage, and enhanced mechanisms of inflammation resolution [32,55,56]. RELB activation by TNF-α induces chromatin remodeling within the granulocyte–macrophage colony-stimulating factor promoter, which downstream switches off canonical NF-κB, driving recovery of homeostasis. Differences in TNF-α concentrations between ISF and conditioned media from treated wells suggest that geraniol and pioglitazone further enhance such a response. Early rises in IL6 gene expression, as observed for treatment with pioglitazone and its combination with geraniol, are desirable in a pro-resolving response and more closely resembled BMNCs at 48 h. Preservation of the IL6 signaling axis is supporting evidence of the difference between anti-inflammation and pro-resolution. IL6 expression is essential in initiating the transcription for the IL4 receptor [57], as well as leading STAT3-mediated IL4 production [58]. The Il-4–STAT6–PPARγ axis drives the expansion of the RXR heterodimer cistrome, providing complex ligand responsiveness in macrophages, which regulates the “epigenomic rachet” of pro-resolving macrophages [38,59]. These changes in RELB and IL6 expression followed the timeline expected for a pro-resolving response as previously reported [27,32,47]. While rises in IL10 are classically expected in anti-inflammatory or pro-resolving responses, IL10 production is directly proportional to increases in TNF-α as an autoregulatory feedback response [54]. Therefore, lower concentrations of TNF-α in the conditioned ISF of the treated groups may explain the ultimately low IL10 concentrations and could suggest better preserved homeostasis.

Considering the changes in the expression of *NRF2* and *PPARGC1A*, both interactive targets of PPARγ during mitochondria biogenesis and tolerance to oxidative stress [60,61,62,63], the lack of pioglitazone and geraniol effects on flow cytometry measurements was unexpected. BMNC treatment resulted in a significant reduction in mitochondrial mass at 48 h and a trend of lower mitochondrial mass and ROS production at all time points. Whether such an observation is due to the smaller BMNCs and BMNC-treated cells emitting less fluorescence or an actual reduction in mitochondrial content/ROS production requires further investigation. Overall, our flow cytometry findings may reflect either a sensitivity limitation of the assay or the need for models with more overt inflammation to evaluate the effects of pioglitazone and geraniol on mitochondrial mass, function, and distinctive types of ROS production [62,64]. It is possible that flow cytometric assessment of samples generated with spiked ISF (LPS + cytokine) would have crossed the sensitivity threshold of the assay.

The improved histological changes in the synovial membrane hemorrhage and vascularity from geraniol-treated joints and cartilage proteoglycan in both geraniol- and pioglitazone-treated joints were consistent with previous reports [35,45]. Even from a small sample, these findings combined with changes observed in vitro for both treatments and their combination (GP) suggest that further in vivo studies with larger samples assessing the effects of both molecules separately and combined are warranted. Moreover, optimization of treatment regimens and delivery methods, such as the use of nanoparticles to enhance drug retention in the joint and hence its effect [65], are required.

Limitations of our study are inherent to the relatively small sample of horses (especially in the in vivo study), which increases the risk of missing subtle effects. Such risks are further increased when using models that do not apply an overt inflammatory stimulus and explore a limited number of predicted outcome measures in discovery research. Nonetheless, our innovative model utilizing primary macrophages cultured in media containing autologous serum and challenged with autologous synovial fluid eliminates artifacts resulting from immune responses to allogeneic or xenogeneic proteins, such as commercial horse serum or FBS. Further, traditional in vitro assays with immune cells use concentrations of cytokines (and LPS) manyfold higher than the limits detected in natural disease. These models do not reflect even the most severe case of naturally occurring joint inflammation [66,67,68]. While the biological variability intrinsic to the response of primary cells, combined with a lower (and more natural) inflammatory stimulus, introduce significant challenges to identify statistically significant differences, our model better translates the inflammatory complexities of OA. In such a scenario, the combined changes in the expression of some PPARγ target genes, along with changes in cytokine concentrations in conditioned media and in vivo findings, suggest that both geraniol and pioglitazone may enhance pro-resolving and chondroprotective mechanisms. Further, only nine genes were carefully selected for gene expression analysis. More comprehensive transcriptomic and lipidomic assessments could provide insights into the pathways affected by these PPARγ agonists that might be missed by assessing selective targets. Ongoing full-transcriptome studies may help elucidate the comparative effects of geraniol and pioglitazone on PPARγ target engagement and their role in macrophage-mediated resolution of synovial inflammation. Functional assays, such as assessment of phagocytosis/efferocytosis or cytokine response upon re-stimulation, would add to our findings, but were beyond the scope of this pilot study.

## 5. Conclusions

This study highlights the potential of the PPARγ agonists geraniol and pioglitazone to modulate the expression of key genes and cytokines with homeostatic and pro-resolving functions in macrophages exposed to inflamed synovial fluid. Notably, the upregulation of RELB and NRF2, alongside the modulation of IL6 and TNF-α, suggests that PPARγ agonism can promote pathways associated with inflammation resolution and mitochondrial function. However, no significant changes were observed in mitochondrial mass or ROS production measured by flow cytometry, highlighting the complexity of these processes and the limitations of this pilot study. Our findings emphasize the therapeutic potential of these PPARγ agonists in facilitating recovery from inflammation and promoting joint homeostasis. Future studies using larger samples and more comprehensive omics analyses are warranted to further address this study’s limitations and explore the therapeutic impact of PPARγ in resolving chronic synovial inflammation and halting the development of OA.

## Data Availability

Data not presented in the body of the article or Supplementary Materials are available upon requests directed to the corresponding author.

## Supplementary Materials

The following supporting information can be downloaded at: https://www.mdpi.com/article/10.3390/biom15091267/s1, **Table S1** Primers and referred sequences used to assess target gene expression; **Supplementary Figure S1** Patterns of expression of PPARG and PPARGC1A in bone marrow-derived macrophages in response to inflamed synovial fluid (ISF) with the addition of geraniol (G), pioglitazone (P), or a combination of both (GP). Macrophages cultured in normal synovial fluid (SF), ISF alone, and ISF + bone marrow mononuclear cells (BMNCs) served as controls. Data were normalized to ISF at each time point. **Supplementary Figure S2** Patterns of expression of NRF1 and NRF2 in bone marrow-derived macrophages in response to inflamed synovial fluid (ISF) with the addition of geraniol (G), pioglitazone (P), or a combination of both (GP). Macrophages cultured in normal synovial fluid (SF), ISF alone, and ISF + bone marrow mononuclear cells (BMNCs) served as controls. Data were normalized to ISF at each time point. **Supplementary Figure S3** Patterns of expression of TFAM and RELB in bone marrow-derived macrophages in response to inflamed synovial fluid (ISF) with the addition of geraniol (G), pioglitazone (P), or a combination of both (GP). Macrophages cultured in normal synovial fluid (SF), ISF alone, and ISF + bone marrow mononuclear cells (BMNCs) served as controls. Data were normalized to ISF at each time point. **Supplementary Figure S4** Patterns of expression of IL10 and IL6 in bone marrow-derived macrophages in response to inflamed synovial fluid (ISF) with the addition of geraniol (G), pioglitazone (P), or a combination of both (GP). Macrophages cultured in normal synovial fluid (SF), ISF alone, and ISF + bone marrow mononuclear cells (BMNCs) served as controls. Data were normalized to ISF at each time point. **Supplementary Figure S5** Patterns of expression of IL1B in bone marrow-derived macrophages in response to inflamed synovial fluid (ISF) with the addition of geraniol (G), pioglitazone (P), or a combination of both (GP). Macrophages cultured in normal synovial fluid (SF), ISF alone, and ISF + bone marrow mononuclear cells (BMNCs) served as controls. Data were normalized to ISF at each time point. **Supplementary Figure S6** Patterns of changes in the concentrations of TNF-α, IL10, and IL1β in macrophage-conditioned inflamed synovial fluid (ISF) with the addition of geraniol (G), pioglitazone (P), or a combination of both (GP). Macrophage-conditioned normal synovial fluid (SF), ISF alone, and ISF + bone marrow mononuclear cells (BMNCs) served as controls. **Supplementary Figure S7** Patterns of changes in the concentrations of IL6 and SDF1 in macrophage-conditioned inflamed synovial fluid (ISF) with the addition of geraniol (G), pioglitazone (P), or a combination of both (GP). Macrophage-conditioned normal synovial fluid (SF), ISF alone, and ISF + bone marrow mononuclear cells (BMNCs) served as controls.

## Abbreviations

The following abbreviations are used in this manuscript:

PPARγ	peroxisome proliferator-activated receptor gamma
OA	osteoarthritis
NSAIDs	non-steroidal anti-inflammatory drugs
*PPARGC1a*	PPARγ coactivator 1A gene
*RELB*	transcription factor RELB gene
*IL6*	interleukin 6 coding gene
IL6	interleukin 6 protein
*NRF2*	interleukin 6 protein
TNF-α	tumor necrosis factor alpha
PGE_2_	prostaglandin E_2_
NF-κB	nuclear factor kappa B
IL10	interleukin-10 protein
IL1β	interleukin-1β protein
IL1α	interleukin-1α protein
IGF1	insulin-like growth factor 1
SDF1	stromal cell-derived factor 1
MCP1	macrophage chemotactic protein 1
BMNCs	bone marrow mononuclear cells
BMDMs	bone marrow-derived macrophages
G	geraniol
P	pioglitazone
GP	geraniol and pioglitazone combined
SF	synovial fluid
ISF	inflamed synovial fluid
PCR	polymerase chain reaction
ROS	reactive oxygen species
LPS	lipopolysaccharide
IU	international units
DPBS	Dulbecco’s phosphate-buffered saline
DMSO	dimethyl sulfoxide
FBS	fetal bovine serum
M-CSF	macrophage-colony stimulating factor
AVMA	American Veterinary Medical Association
mRNA	messenger ribonucleic acid
cDNA	complementary deoxyribonucleic acid
MdFI	median fluorescence intensity
MinDC	minimum detectable concentration
AZF	acetic zinc formalin
OARSI	Osteoarthritis Research Society International
LLOD	lower limit of detection
ANOVA	analysis of variance
H&E	hematoxylin and eosin
Saf-O	safranin O
RXR	retinoid X receptor
