# Supplementary material for: PPARγ Agonism Modulates Synovial Macrophage and Cartilage Responses in an Equine Model of Synovial Inflammation—Implications for Joint Therapy
Source: Biomolecules. 2025 Sep 1;15(9):1267. doi: 10.3390/biom15091267 (PMC12467806; doi:10.3390/biom15091267)
Supplement: Supplementary file 1 [file biomolecules-15-01267-s001.zip › biomolecules-3717635-supplementary.pdf]

Supplementary Table S1 Primers and referred sequences used to assess target gene expression

**Target**	Accession #	Forward primer (5′-3′)	Reverse Primer (5′-3′)	Product size (bp)
RELB	XM_023649886.1	CCCGTCTACGACAAGAAGTCC	TTGTCGCACAGCAGGTAGAG	110
Il10	NM_001082490.1	CTGCCCCACATGCTCCAT	AGCTGGTCCTTCATTTGAAAGAA	74
IL1B	XM_001495926.4	CACAGGCCTTCCAGGATGA	TGTCACAGATGATGGGTTCTTCTT	75
IL6	NM_001082496.2	GGATGCTTCCAATCTGGGTTCAAT	TCCGAAAGACCAGTGGTGATTTT	65
PPARG	XM_001492411.5	TGCTCCACACTATGAAGACA	GCAGGCTCCACTTTGATCGC	108
PPARGC1a	XM_001499929.5	TGTGCAACCAGGACTCTGTA	ACTTGAGTCCACCCAGAAAGC	150
NRF1	XM_023639670.1	GTGGTCCAGACCTTTAGTAACC	CCATCAGCCACAGCAGAATA	146
NRF2	XM_014736323.2	GCCTTACCGGAAGAGACACT	GCTCCTCCGCTTCTCTTTTAGT	150
TFAM	XM_023643450.1	CTCAGAACCCAGATGCGAAA	CTGCCCTGTAAGCATCTTCATA	108
ACTB	NM_001081838.1	CGACATCCGTAAGGACCTGT	CAGGGCTGTGATCTCCTTCT	100
GAPDH	NM_001163856.1	CAAGTTCCATGGCACAGTCAAG	GGCCTTTCCGTTGATGACAA	58

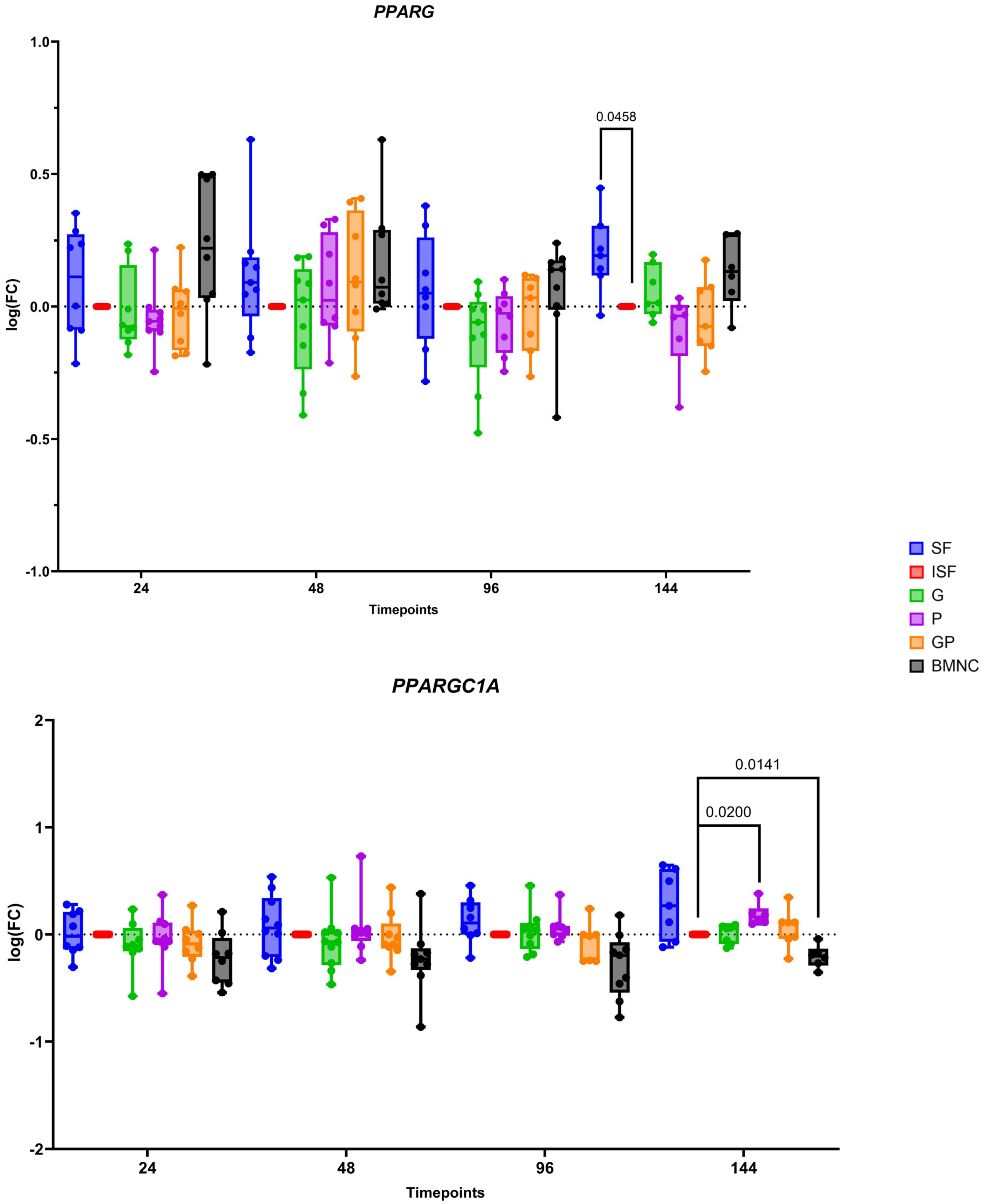

Supplementary Figure S1: Patterns of expression of PPARG and PPARGC1A in bone marrow-derived macrophages in re-sponse to inflamed synovial fluid (ISF) with the addition of geraniol (G), pioglitazone (P), or a combination of both (GP). Macrophages cultured in normal synovial fluid (SF), ISF alone, and ISF + bone marrow mononuclear cells (BMNCs) served as controls. Data were normalized to ISF at each time point.

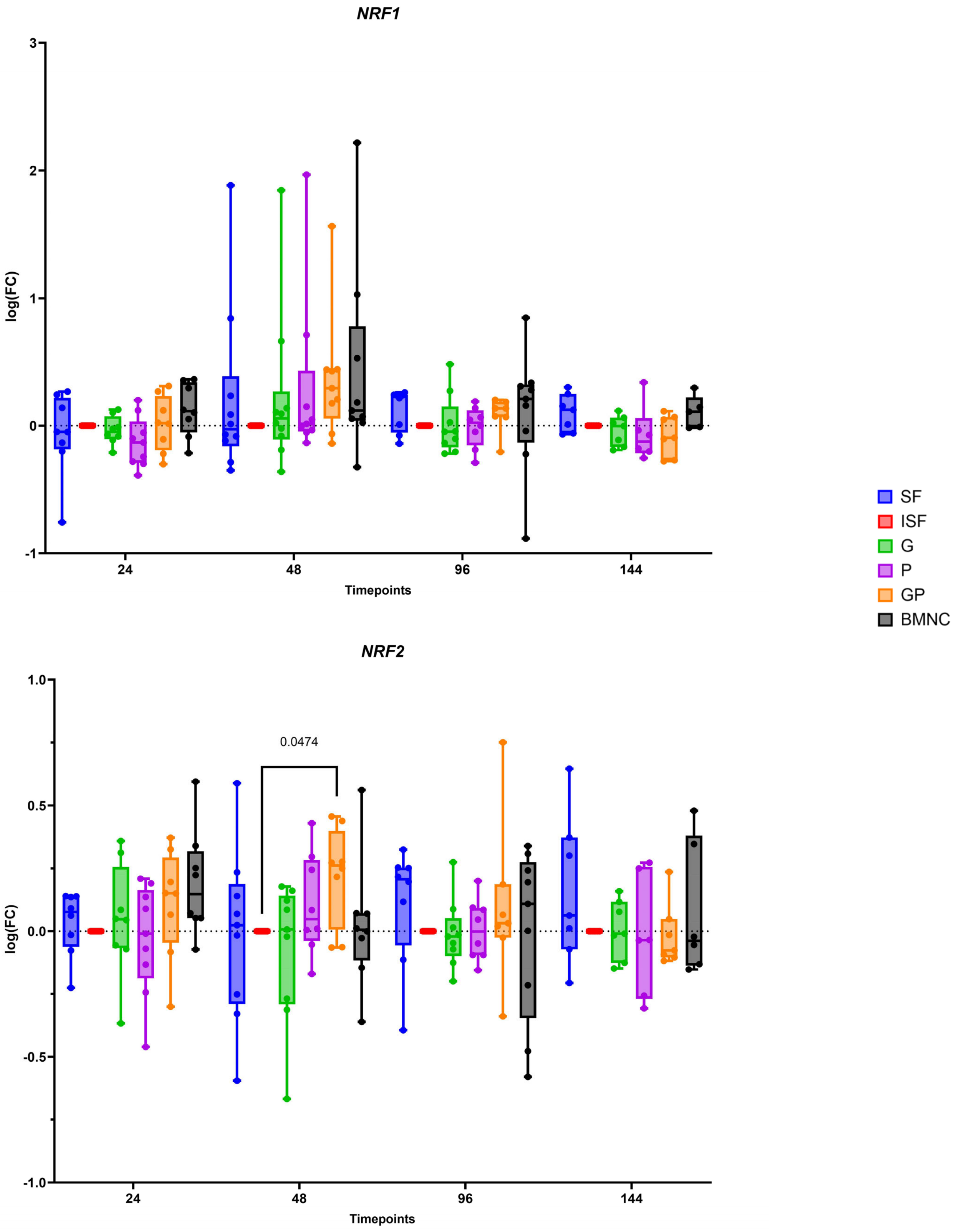

Supplementary Figure S2: Patterns of expression of NRF1 and NRF2 in bone marrow-derived macrophages in response to inflamed synovial fluid (ISF) with the addition of geraniol (G), pioglitazone (P), or a combination of both (GP). Macrophages cultured in normal synovial fluid (SF), ISF alone, and ISF + bone marrow mononuclear cells (BMNCs) served as controls. Data were normalized to ISF at each time point

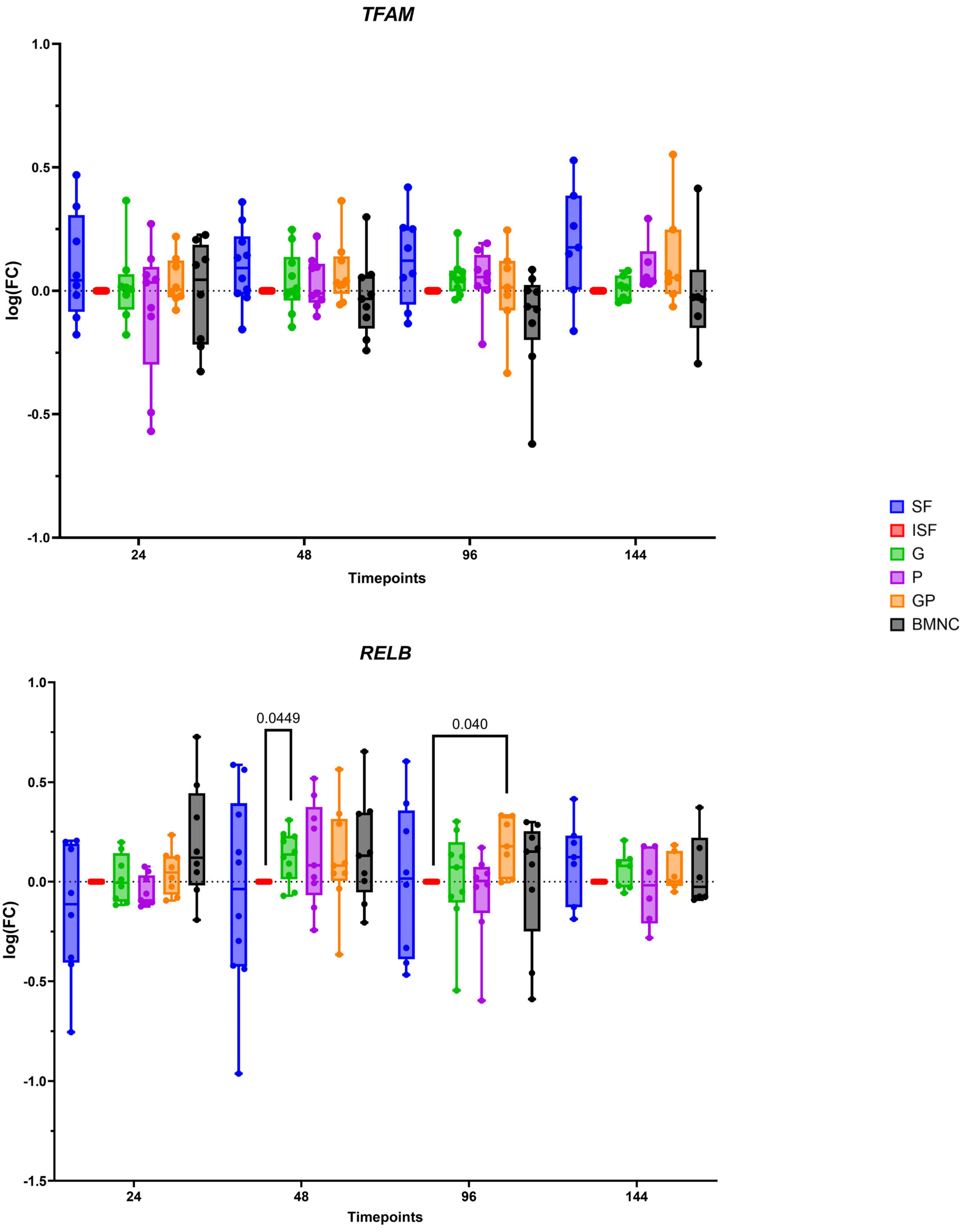

Supplementary Figure S3: Patterns of expression of TFAM and RELB in bone marrow-derived macrophages in response to inflamed synovial fluid (ISF) with the addition of geraniol (G), pioglitazone (P), or a combination of both (GP). Macrophages cultured in normal synovial fluid (SF), ISF alone, and ISF + bone marrow mononuclear cells (BMNCs) served as controls. Data were normalized to ISF at each time point.

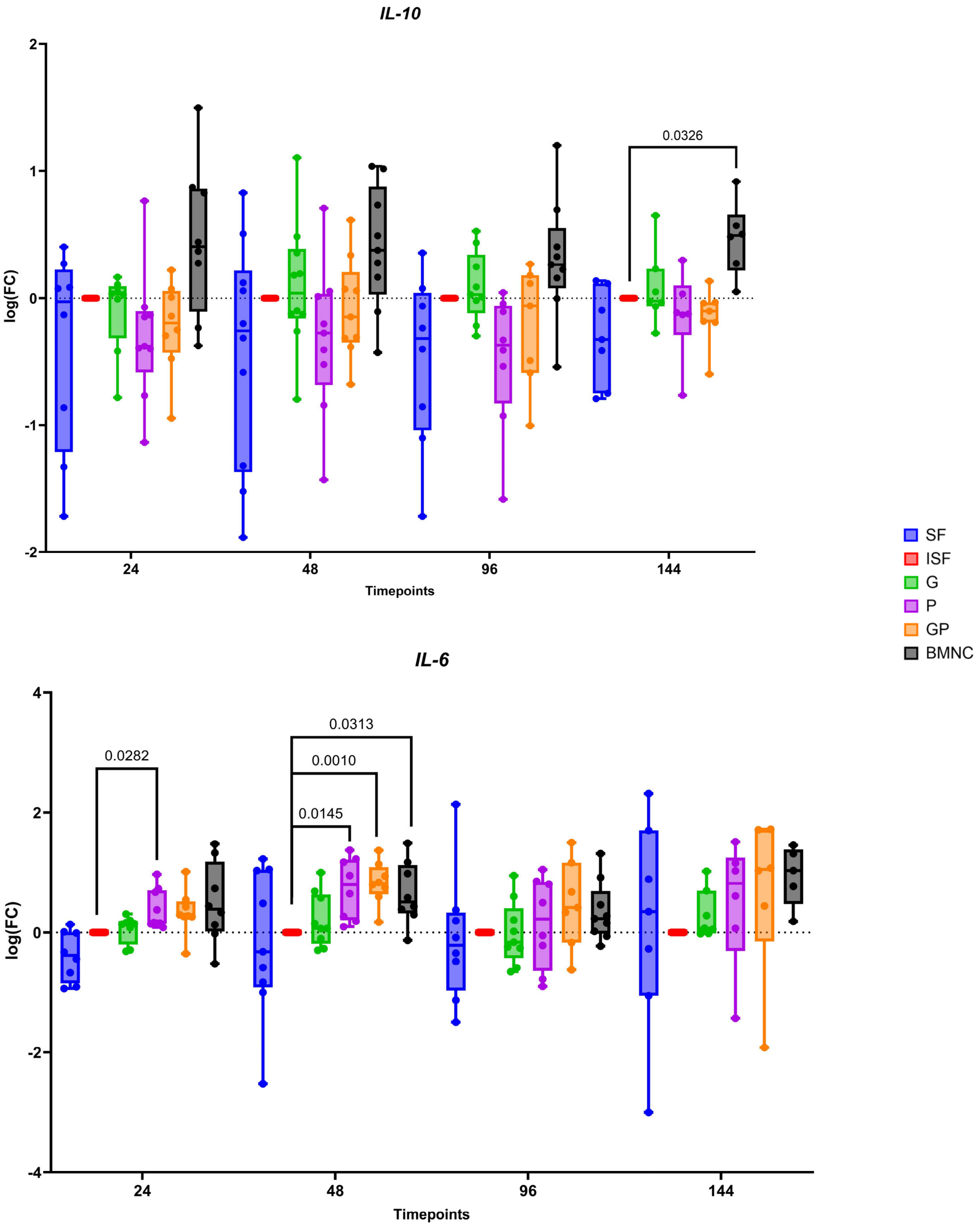

Supplementary Figure S4: Patterns of expression of IL10 and IL6 in bone marrow-derived macrophages in response to in-flamed synovial fluid (ISF) with the addition of geraniol (G), pioglitazone (P), or a combination of both (GP). Macrophages cultured in normal synovial fluid (SF), ISF alone, and ISF + bone marrow mononuclear cells (BMNCs) served as controls. Data were normalized to ISF at each time point.

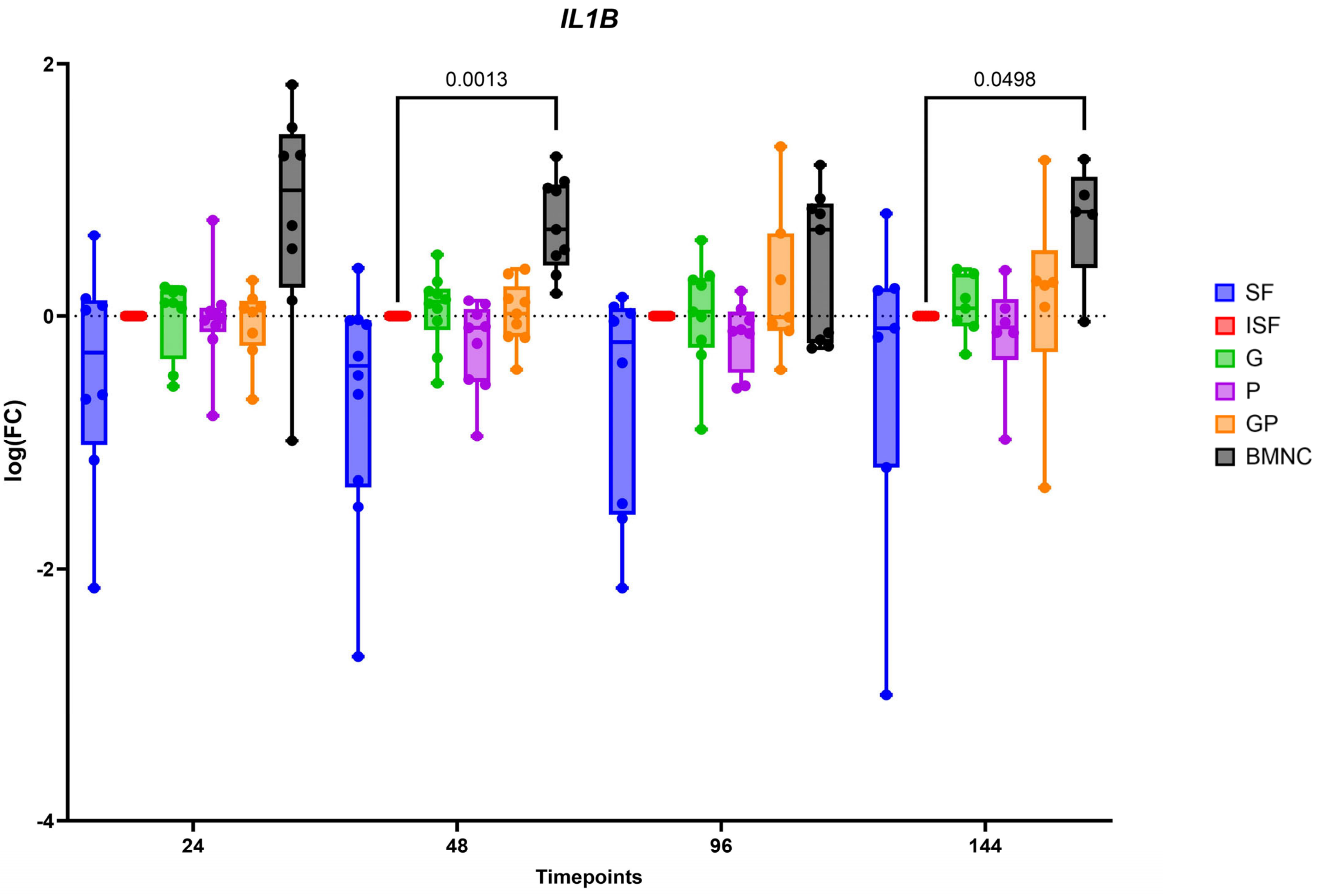

Supplementary Figure S5: Patterns of expression of IL1B in bone marrow-derived macrophages in response to inflamed syn-ovial fluid (ISF) with the addition of geraniol (G), pioglitazone (P), or a combination of both (GP). Macrophages cultured in normal synovial fluid (SF), ISF alone, and ISF + bone marrow mononuclear cells (BMNCs) served as controls. Data were normalized to ISF at each time point.

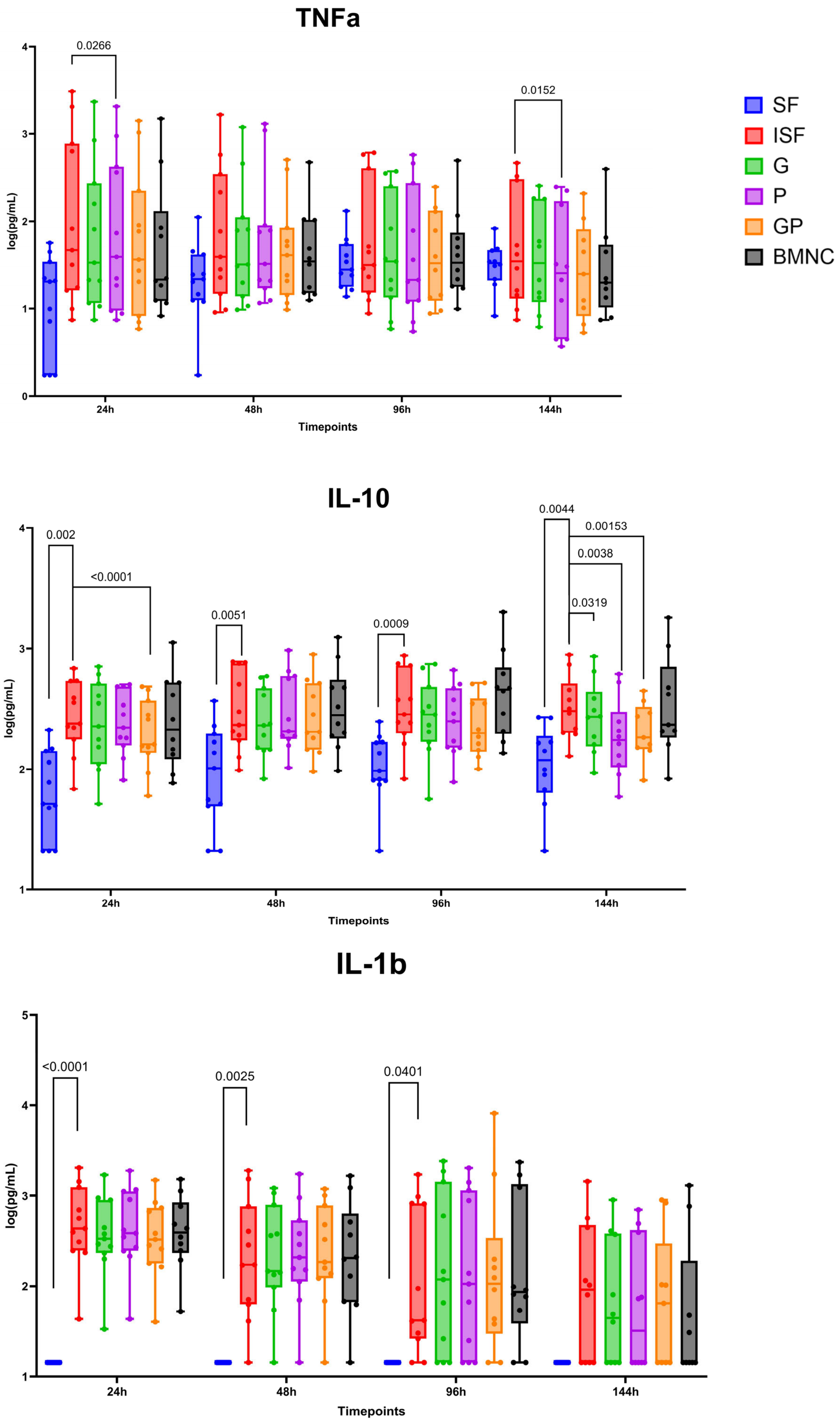

Supplementary Figure S6: Patterns of changes in the concentrations of TNF-α, IL10, and IL1β in macrophage-conditioned inflamed synovial fluid (ISF) with the addition of geraniol (G), pioglitazone (P), or a combination of both (GP). Macrophage-conditioned normal synovial fluid (SF), ISF alone, and ISF + bone marrow mononuclear cells (BMNCs) served as controls.

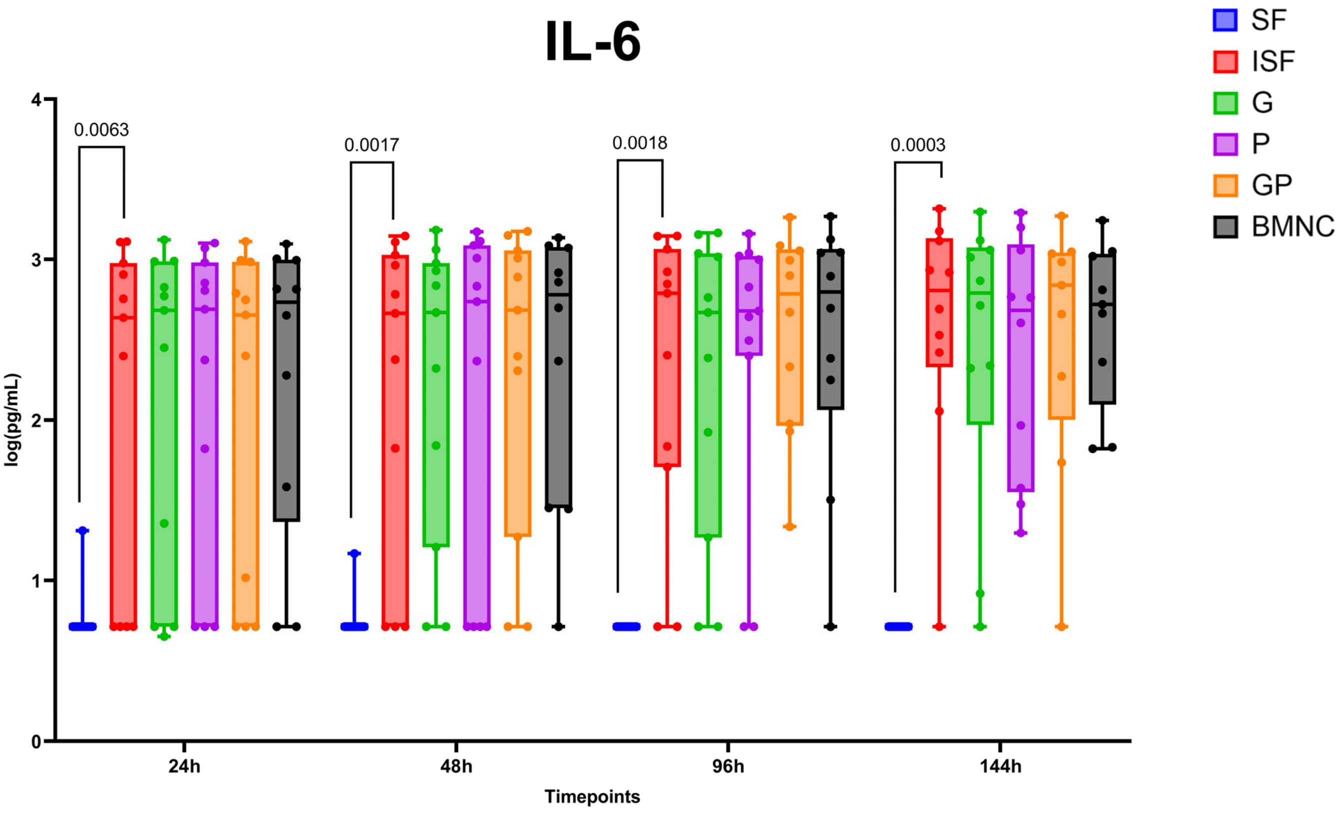

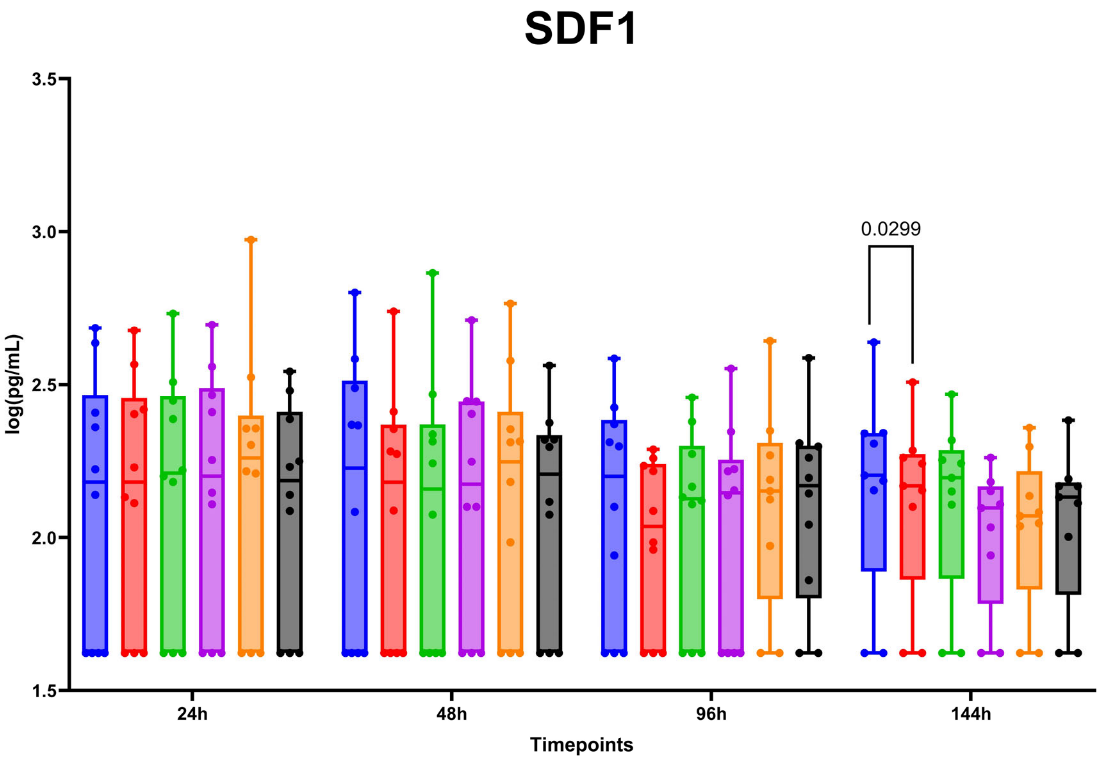

Supplementary Figure S7: Patterns of changes in the concentrations of IL6 and SDF1 in macrophage-conditioned inflamed synovial fluid (ISF) with the addition of geraniol (G), pioglitazone (P), or a combination of both (GP). Macrophage-conditioned normal synovial fluid (SF), ISF alone, and ISF + bone marrow mononu-clear cells (BMNCs) served as controls.
